# The Use of ^99m^Tc-Methoxy-isobutyl-isonitrile (sestaMIBI) Uptake on Scintigraphy (^99m^-STS) in Amiodarone-Induced Thyrotoxicosis: Case Series and Review of the Literature

**DOI:** 10.1155/2020/2493460

**Published:** 2020-08-01

**Authors:** Ghada Elshimy, Mahmoud Alsayed, Jerome Targovnik, Gamal Sidarous, Kresimira M. Milas

**Affiliations:** ^1^Endocrinology, Diabetes and Metabolism Division, Medical College of Georgia, Augusta University, Augusta, GA, USA; ^2^Endocrinology Division, University of Arizona College of Medicine, Phoenix, AZ, USA; ^3^Endocrinology Division, Phoenix VA Health Care System, Phoenix, AZ, USA; ^4^Nuclear Medicine Department, University of Arizona College of Medicine, Phoenix, AZ, USA; ^5^Endocrine Surgery Department, University of Arizona College of Medicine, Phoenix, AZ, USA

## Abstract

Amiodarone is a class III antiarrhythmic drug, used by cardiologists to treat arrhythmia including atrial fibrillation (A fib) and ventricular fibrillation. However, amiodarone is associated with endocrine dysfunction including both hypo- and hyperthyroidism. In the literature, two types of amiodarone-induced thyrotoxicosis (AIT) were described: AIT-1 and AIT-2. Mixed AIT also called AIT type 3 (AIT-3) has been described in the literature when the cases do not have a typical presentation. In order to differentiate different types of AIT, various clinical, biochemical, and radiological tools have been proposed. The use of ^99m^Tc-methoxy-isobutyl-isonitrile (sestaMIBI) uptake on scintigraphy (^99m^-STS) has been suggested in the literature in only few studies (no large retrospective or prospective studies have been established in the United States). We present a case series describing 5 patients presenting to the University of Arizona with AIT where we used ^99m^-STS to assess in diagnosis and treatment of different types of AIT followed by a review of the literature.

## 1. Introduction

Amiodarone is a class III antiarrhythmic drug, used by cardiologists to treat arrhythmia including atrial fibrillation (A fib) and ventricular fibrillation. However, amiodarone is associated with endocrine dysfunction including both hypo- and hyperthyroidism. This dysfunction is explained by the high iodine content (containing 75 mg of iodine per 200 mg tablet) and its direct toxic effect on the thyroid [1]. Amiodarone principle metabolite is desethylamiodarone (DEA), which accumulates in different tissues including the lung, the adipose tissues, and the thyroid. Given the very long elimination half-lives of amiodarone and DEA (40 ± 10 days and 57 ± 27 days, respectively), the drug and its metabolites remain available for a long time after withdrawal of the medication [2].

The intrinsic properties of the drug explain the effect of amiodarone on thyroid hormone. It inhibits the outer ring 5'-monodeiodination of thyroxine (T4). Additionally, it inhibits the intracellular T4 transport and pituitary type 2 idothyronine deiodinase (D2), thus leading to a decrease in the triiodothyronine (T3) production with an increase in the reverse T3 level. Furthermore, DEA blocks T3-receptor binding to nuclear receptors and decreases the expression of some thyroid hormone-related genes. In addition, there is a direct toxic effect on the thyroid follicular cells, resulting in a destructive thyroiditis referred to amiodarone-induced thyrotoxicosis type 2 (AIT-2). The iodine content in amiodarone mediates amiodarone-induced thyrotoxicosis type 1 (AIT-1) and amiodarone-induced hypothyroidism (AIH). AIT-1 has underlying abnormal thyroid gland (nodular goiter and latent Graves' disease) with positive antibody testing which explains the pathophysiology of the disease [3–6].

Amiodarone can have additional effects on the thyroid function tests. There is a transient increase in thyroid-stimulating hormone (TSH) in the first months of treatment in nearly all patients treated with amiodarone given the Wolff–Chaikoff effect explained by the adaptation of the thyroid gland to iodine overload with inhibition of iodine organification and reduction of the thyroid hormones production rate [3–6].

In the literature, two types of amiodarone-induced thyrotoxicosis (AIT) were described: AIT-1 and AIT-2. Mixed AIT also called AIT type 3 (AIT-3) has been described in the literature when the cases do not have a typical presentation. Some authors further divided the mixed AIT-3 form into mixed AIT/type 1 and mixed AIT/type 2 [7, 8]. In order to differentiate different types of AIT, various clinical, biochemical, and radiological tools have been proposed. On clinical examination, the presence of Graves' disease or Graves' ophthalmopathy or goiter points toward AIT-1. Laboratory testing as measurement of the inflammatory mediators has been suggested. Erythrocyte sedimentation rate (ESR) and C-reactive protein (CRP) can be elevated in AIT-2 in the context of thyroiditis, while interleukin-6 (IL-6) is considered a better marker for the destructive process. Unfortunately, sometimes, it can be falsely low, which limits its usefulness. In addition, coexisting illness such as heart failure or preexisting thyroid disease like Graves' disease can produce high IL-6 making it an unreliable marker in differentiating between AIT types [4, 9–11]. Multiple imaging modalities have been suggested including color flow Doppler sonography (CFDS) and radioactive iodine uptake scan (RAIU). Absent vascularity on CFDS is seen in patients with AIT-2, while uneven patchy parenchymal flow to markedly diffuse increase in the flow is seen in AIT-1. Studies comparing both modalities showed that CFDS is more accurate, but combined modalities have been suggested for more accurate results [12–14]. Moreover, differences in populations' iodine supply have often been mentioned as one of the main reasons for the poor reproducibility of CFDS and RAIU [15, 16].

In the past couple of years, the use of ^99m^Tc-methoxy-isobutyl-isonitrile (sestaMIBI) uptake on scintigraphy (^99m^-STS) has been suggested in the literature in only few studies to differentiate between different types of AIT (no large retrospective or prospective studies have been established in the United States). The rationale behind using ^99m^-STS is that given the short half-life of the pure gamma emitter tracer Tc-99m, it has ability to accumulate in the mitochondrial rich cells, and it can differentiate between AIT-1 and AIT-2. There is positive uptake in hyperfunctioning thyroid in AIT-1, and no uptake is observed in AIT-2. The duration of the test is shorter in comparison to RAIU, and the results are not influenced by the iodine supply in the population making it a more promising diagnostic tool. In 2008, Piga et al. used it to distinguish AIT-1 from AIT-2 in Italy and stated that it is an effective tool superior to CFDS and RAIU [17]. In 2015, Pattison et al. described 15 patients in Australia, and in 2017, Wang and Zhang described 15 patients in China where they used ^99m^-STS [18, 19]. In 2018, Censi et al. used ^99m^-STS with target to background ratio (TBR) on 30 patients with AIT in Italy and suggested that ^99m^-STS has 100% specificity and 91.7% sensitivity in differentiating different types of AIT [8]. We present a case series describing 5 patients presenting to the University of Arizona with AIT where we used ^99m^-STS to assess in diagnosis and treatment of different types of AIT followed by a review of the literature.

## 2. Materials and Methods

In the case series, ^99m^-STS was performed in our facility at the University of Arizona for all the described patients. It is a dual-head gamma camera equipped with pinhole collimators (Siemens, Symbia Evos). Images were acquired after intravenous injection of about 10–25 mCi of sestaMIBI. Early images were acquired at 10–15 minutes, and the late images were acquired at 60–180 minutes in different patients. Most of the prior reports acquired late images at 60 minutes; however, since this is a new modality used for AIT, the nuclear medicine department suggested extending the duration up to 180 minutes to assess if it will have an effect on the diagnosis, to ensure the complete washout of the tracer without any residuals and to rule out any mixed AIT pattern.

A qualitative assessment was based on the pattern of tracer uptake: diffuse tracer retention in the early images and a complete washout of the radiopharmaceutical in the late images prompted the patient to be classified as a case of AIT-1; if there was no significant tracer uptake in the early images, the patient was diagnosed as having AIT-2. This is similar to Piga et al. in 2008 and Censi et al. in 2018 description for the use of 99m-STS in AIT patients. Regarding the mixed AIT (AIT-3) pattern, it was suggested to use the description of Censi et al., which further classified AIT-3 into mixed/AIT-1 and mixed/AIT-2. Mixed/AIT-1 was diagnosed if the scan had a normal or slightly lower uptake than in AIT-1 in the early images and only a partial washout in the late images. Mixed/AIT-2 was diagnosed if the scan showed a slightly higher uptake than in AIT-2 in the early images and partial or complete washout in the late ones [8, 17]. However, none of our patients had a mixed pattern.

The laboratory reference ranges throughout the manuscript were as follow: TSH: 0.45–5 mIU/L; free T4: 0.8–1.7 ng/dl; free T3: 2–4.8 pg/ml; thyroid-stimulating immunoglobulin (TSI): <140%; TSH receptor antibodies (TRAb): <OR = 16%, microsomal thyroid peroxidase (TPO) antibody: ≤34 IU/ml; and 24 hours urine iodine: 70–500 mcg/24 h.

## 3. Cases Description

### 3.1. Patient 1

**Figure 1:**
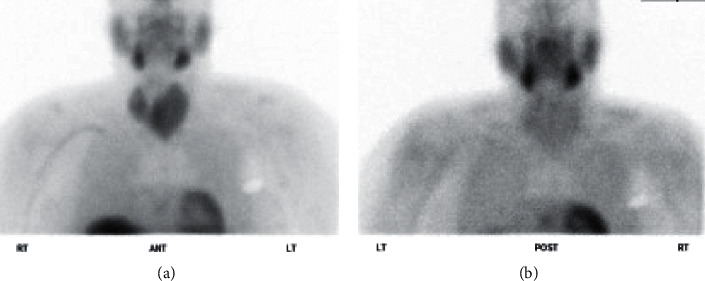
The ^99m^-STS with a dose of 21.8 mCi showed intense initial uptake of the tracer activity within the thyroid parenchyma followed by a near complete washout in the 3-hour delayed images suggestive of AIT-1. In addition, there is diffuse enlargement of the thyroid gland (left more than right) and physiologic pattern of tracer distribution observed in the nasopharyngeal mucosa, salivary glands, and imaged parts of the liver and myocardium. A photopenic area in the left chest region correlates with the pacemaker. (a) 10 min postinjection; (b) 3 hr postinjection.

A 69-year-old male with a history of type 2 diabetes, hypertension, multinodular goiter (MNG) without compressive symptoms, and A fib was referred to our endocrine clinic given abnormal thyroid function tests. He had an automatic implantable cardioverter-defibrillator (AICD) implant, and he has been on amiodarone for almost 12 months to control his A fib. He denied any symptoms of hyperthyroidism. He was following with his cardiologist, and laboratory tests showed suppressed TSH <0.01 mU/l with high normal free T4 1.7 ng/dl, 6 months after starting the amiodarone. There was no documented baseline TSH prior to the amiodarone use. Repeat laboratory workup showed normal free T4 1.3 ng/dl and normal free T3 3.1 pg/ml with TSH <0.01 mU/l. Antibodies testing showed TSI <89%, TRAb 7%, and TPO antibody 31 IU/mL. A 24-hour urine iodine was ordered and came back elevated >1000 mcg/L, so ^99m^-STS was ordered instead of RAIU to assess for AIT. The ^99m^-STS showed intense initial intense uptake of the tracer activity within the thyroid parenchyma followed by a near complete washout in the 3-hour delayed images suggestive of AIT-1 (Figure 1). Thyroid ultrasound (US) showed diffusely enlarged (left more than right) thyroid gland with relatively isoechoic, predominantly solid nodules replacing the entire gland. Right lobe has dominant midupper pole nodule 3.7 × 1.9 × 2.2 cm. Two lower pole nodules measure 2.6 × 1.2 × 1.3 cm and 1.9 × 1.3 × 1.9 cm. Left lobe contains a dominant nodule measuring 7.1 × 5.1 × 5.7 cm. There was mild diffuse internal blood flow on CFDS. The patient was started on methimazole 10 mg tablets orally (PO) daily, and he was referred to endocrine surgery for total thyroidectomy given the large-size goiter and the worsening underlying cardiac condition. Pathology revealed multinodular colloid goiter with no evidence of malignancy. Afterward, thyroid function tests normalized. Amiodarone was continued to control his A fib.

### 3.2. Patient 2

**Figure 2:**
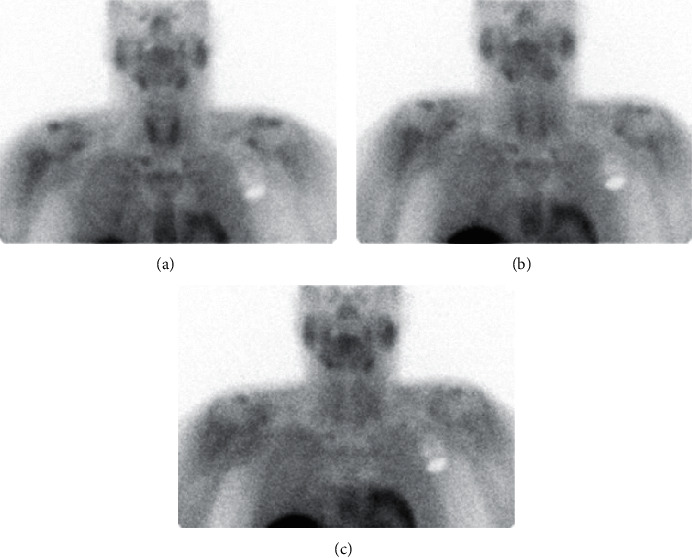
The ^99m^-STS with a dose of 19.4 mCi showed intense initial intense uptake of the tracer activity within the thyroid parenchyma followed by bilateral symmetrical gradual washout of tracer activity in the 1-hour and 2-hour delayed images suggestive of AIT-1. (a) 15 min delay; (b) 1 hr delay; (c) 2 hr delay.

A 62-year-old male with a history of nonischemic cardiomyopathy and recurrent ventricular tachycardia (VT) status post-AICD implant 2 years before was admitted to an outside hospital with recurrent persistent VT. The patient was on amiodarone 200 mg PO daily for 1 year and then he was switched to sotalol around 10 months prior to the presentation. Baseline TSH was normal prior to the start of amiodarone. Laboratory workup showed free T4 2.83 ng/dl and TSH <0.005 mU/L. Thyroid US showed mild diffuse enlargement with no nodules. Methimazole 20 mg 4 times PO daily with metoprolol tartrate 100 mg twice PO daily were started without any further workup with a presumptive diagnosis of AIT-1. The patient presented to our hospital 3 months later with recurrent VT. Repeat laboratory workup showed free T4 5.06 ng/dl, TSH <0.01 mU/L, free T3 6 pg/ml with negative antibodies testing, TSI 113%, and TRAb 2%. The patient stated that he was compliant to his medications. Given the persistent hyperthyroidism despite high-dose methimazole, ^99m^-STS was ordered. The ^99m^-STS showed similar results to patient 1, suggesting AIT-1 (Figure 2). Methimazole was discontinued, and propylthiouracil 200 mg every 6 hours was started. After 10 days, repeat free T4 showed significant improvement (2.2 ng/dl) with normalization of free T3 at 3 pg/ml.

### 3.3. Patient 3

**Figure 3:**
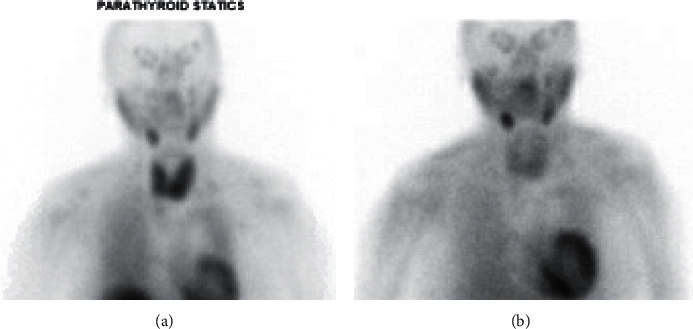
^99m^-STS with a dose of 19.43 mCi showed an intense initial intense uptake of the tracer activity within both thyroid lobes (a) followed by a complete washout in the 2-hour delayed images (b) suggestive of AIT-1.

A 55-year-old female with a history of end-stage renal disease secondary to Goodpasture syndrome, secondary hyperparathyroidism, osteoporosis, MNG with multiple benign fine-needle aspiration (FNA) of different nodules, and amiodarone use for VT 100 mg daily for 1 year was referred to the endocrine clinic given symptoms of hyperthyroidism including weight loss, recurrent palpitations, tremors, and heat intolerance. Laboratory workup showed suppressed TSH 0.06 mU/L, high free T4 2.3 ng/dl, and normal free T3 2.6 pg/ml. Negative antibodies testing showed the following values: microsomal TPO antibody <10, TSI <89%, and TRAb <1%. Thyroid US showed enlarged multinodular goiter with normal vascularity of the thyroid gland on CFDS. The ^99m^-STS showed intense initial intense uptake of the tracer activity within both thyroid lobes followed by a complete washout in the 2-hour delayed images suggestive of AIT-1 as observed in patients 1 and 2 (Figure 3). Methimazole 10 mg PO daily was started, and metoprolol 50 mg PO twice daily previously prescribed by the cardiologist was continued. Repeat thyroid function tests 2 months later showed normalization of free T4 and TSH. The patient continued to be on amiodarone to control the VT.

### 3.4. Patient 4

**Figure 4:**
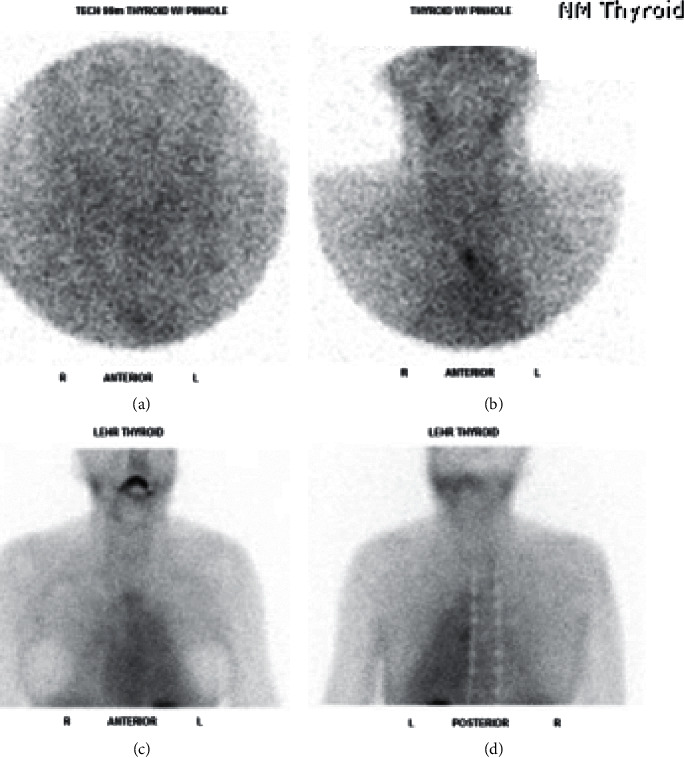
^99m^-STS showed failure of localization of the tracer within the thyroid gland, by imaging criteria suggesting destructive effect of amiodarone to the function of the gland and confirming AIT-2 (a, b) 15 minutes delay high- and low-resolution images. (c, d) 1 hour delayed anterior and posterior images.

A 75-year-old female with a past medical history of persistent A Fib on amiodarone for 2 years that was stopped 5 months earlier presented to the emergency department with recurrent A fib and underwent ablation. She complained of recurrent palpitation with weight loss around 10 pounds in the last month despite having a very large appetite. She had a family history of Hashimoto's hypothyroidism in her father. Laboratory workup showed suppressed TSH 0.01 mU/L, slightly elevated free T4 1.75 ng/dl, normal free T3 3.2 pg/ml, mildly positive TSI 159% and negative TRAb <9 IU/l. Two months prior to the presentation, TSH was normal (1.85 mU/l). The patient had a computerized tomography angiogram with contrast 1 month prior to this presentation ordered by the cardiologist. Decision was to order ^99m^-STS to assess for AIT. It showed no evidence of tracer labeling within the thyroid lobes at the initial 15 minutes' images and the 1-hour follow-up image confirming AIT-2 (Figure 4). Prednisone 40 mg PO daily was started leading to a significant improvement in the patient's symptoms. Prednisone was tapered off over a couple of months with a complete normalization of the thyroid function tests.

### 3.5. Patient 5

**Figure 5:**
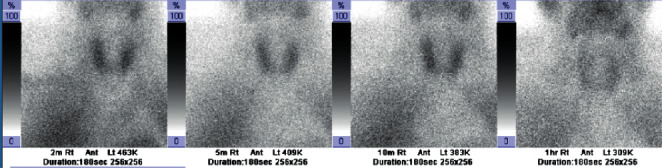
^99m^-STS with a dose of 24.9 mCi demonstrating persistent activity in the right and left lobes of the slightly enlarged thyroid gland at 10 minutes, declining at 60 minutes' image confirming diagnosis of AIT-1.

A 69-year-old male with a past medical history of A fib, type 2 diabetes, hypertension, and heart failure was admitted for recurrent A fib with rapid ventricular rate. Endocrinology service was consulted for suppressed TSH. He has been on amiodarone for 18 months prior to this admission. He complained of tremors and palpitations but no other symptoms related to hyperthyroidism. Physical examination was significant for resting tremors with a palpable mild goiter, but without discrete thyroid nodule or bruit. Laboratory testing showed TSH <0.004 uIU/mL, free T4 3.4 ng/dL, free T3 5.72 pg/mL, negative antibodies with TSI <89%, and TRAb <0.9 IU/L. Thyroid US showed mildly homogenously enlarged thyroid gland. CFDS showed absent vascularity. Based on the lack of underlying thyroid pathology and the absence of vascularity on US, prednisone 40 mg PO daily was started with a presumptive diagnosis of AIT-2. Given no improvement in the patient's clinical condition, repeated thyroid function tests were ordered, and it showed persistent hyperthyroid status with TSH <0.004, free T4 2.18, free T3 2.1, and 24 hours' urine iodine 2905 *μ*g (100–460 ug/24 hours). ^99m^-STS showed persistent activity in the right and left lobes of the slightly enlarged thyroid gland at 10 minutes, unchanged from the 2-minute image and declining at 60 minutes' compatible with AIT-1 (Figure 5). Prednisone dose was tapered, and methimazole 20 mg daily was initiated. Over the 4-month period, he was on methimazole, and the patient was demonstrating clinical and biochemical improvement with normalization of thyroid function tests. Amiodarone was continued as per cardiology recommendation.  Table 1 summarize the 5 cases.

## 4. Discussion

Amiodarone is an iodine-rich antiarrhythmic agent used by cardiologists in the treatment of multiple conditions. Treated patients invariably have underlying heart diseases, which can deteriorate in the setting of hyperthyroidism secondary to AIT raising a concern of an increase in these patient's morbidity and mortality. Given the pharmacological drug composition, around 15–20% of the patients on amiodarone will develop thyroid dysfunction including either AIT or AIH. The iodine intake plays a key role in developing amiodarone-induced thyroid dysfunction. This explains the increased prevalence of AIH in iodine-replete and AIT in iodine-deficient geographical area [1, 15]. The pathophysiology of AIT-1 is explained by an excessive thyroidal hormone synthesis and release induced by the iodine load in patients with underlying thyroid autonomy (nodular or diffuse goiter or latent Graves' disease). This iodide-induced thyrotoxicosis is an example of the Jod–Basedow phenomenon seen in patients with endemic iodine-deficient goiter who are given iodide replacement, thus explaining why thyrotoxicosis is more common in iodine depleted areas. However, AIT-2 is a consequence of a destructive process in patients with normal thyroid gland [3–6]. The differentiation between AIT-1 and AIT-2 is an important prerequisite for the correct therapeutic choice to avoid unnecessary use of combined medications; thus, different modalities have been used including clinical, biochemical, and radiological imaging (Table 2).

The presence of thyroid antibodies can be misleading. Positive TRAb, TPO, or thyroglobulin antibodies usually point toward AIT-1 as they indicate underlying thyroid autoimmune disease. However, the presence of positive titers does not rule out AIT-2. Therefore, further imaging studies are required for the complete workup of AIT in these patients [15, 16, 19, 20]. This has occurred in patient 4 who had a mildly positive TSI 159% (reference 140%) in the setting of positive family history of Hashimotos' hypothyroidism in his father; however, the ^99m-^STS showed AIT-2 with a good response to prednisone 40 mg. We should keep in consideration that these antibodies can be positive in patients with a family history of autoimmune disease, and these patients need a close follow-up. Regarding the other 4 patients, all of them had negative antibody titers which was not helpful in the diagnosis. Thyroid US in patients 1 and 3 showed multinodular goiter, while patients 2 and 5 showed only mild diffuse enlargement of the thyroid. As explained before, the patients with MNG have a higher risk for AIT-1. CFDS can also be misleading sometimes as in patient 5. It showed absent vascularity which led to the diagnosis of AIT-2. Given no response to the treatment, ^99m-^STS was ordered which was able to give an accurate diagnosis. To sum up, the ^99m-^STS was a helpful tool in confirming the diagnosis in all of these patients.

Upon review of the literature, initially, in 2008, ^99m^-STS was described by Piga et al. in a series of 20 patients as being superior to all other diagnostic tests for differentiating AIT subtypes and, in particular, is the only test capable of prospectively identifying AIT [17]. The rationale behind the use of ^99m^-STS lies in that sestaMIBI is accumulated by mitochondria-rich cells, whereas necrotic and apoptotic tissues are unable to take up the sestaMIBI tracer because their mitochondrial membrane potential has collapsed. Hence, this explained the increased retention in hyperfunctioning thyroid tissue as a result of increased mitochondrial numbers in hypermetabolic cells in AIT-1 and the absent of tracer uptake in AIT-2. When a qualitative assessment of the ^99m^-STS image is clearly compatible with AIT-1 or AIT-2, the diagnosis is extremely accurate, and patients can be treated accordingly. Pattison et al. in 2015 reported that the use of quantitative TBR improves the interobserver reliability of reporting ^99m^-STS for investigation of different types of AIT [19]. When the qualitative assessment suggests a mixed form, Censi et al. in 2018 recommended applying the TBR: a value below the cut-off of 0.482 is indicative of AIT-2, while a higher value suggests a genuine mixed type of thyrotoxicosis [8]. Few case reports and studies have been documented in the literature regarding the utility of ^99m^-STS in AIT, but no large prospective studies have been established yet [21–24] (Table 3).

There are some advantages of ^99m^-STS in comparison to CFDS and RAIU. Differences in populations' iodine supply have often been mentioned as one of the main reasons for the poor reproducibility of CFDS and RAIU [15, 16]. However, prior studies showed that ^99m^-STS results were not affected by different geographical distributions in either iodine-deficient areas (like Censi et al. in north east Italy) or iodine sufficient areas (like Piga et al. in Sardinia Italy and Wang et al. in China) [8, 17, 18]. The short duration of the test (1 hour in comparison to 24 hours needed in RAIU) and the short half-life of the pure gamma emitter tracer Tc-99m (6 hours) make ^99m^-STS a superior diagnostic study in comparison to the conventional methods used for the diagnosis of AIT. Additionally, Tc-99m is much safer than the gamma and beta emitter I 131 used in RAIU scintigraphy [8].

AIT-2 is usually a self-limited disease, and patients become euthyroid in 3–5 months. However, given the underlying cardiac disease and the fear of the deterioration of the clinical condition especially in elder population and in patients with left ventricular dysfunction, early treatment to achieve euthyroidism is usually required. Prednisone is considered the most effective treatment in AIT-2 given the anti-inflammatory and membrane stabilization effect. However, thionamides, with or without potassium or sodium perchlorate, are used in AIT-1 in addition to beta-blockers for heart rate and adrenergic symptoms control. The mechanism of action of these medications is to block the thyroid hormones production and deplete the intrathyroidal iodine stores in patients with underlying thyroid diseases. These medications are ineffective in AIT-2; however, they can be used in combination with steroids in AIT-3 (mixed AIT) when the diagnosis is inconclusive [2, 7]. We described different patients with different scenarios. Interestingly, patient 2 was resistant to methimazole treatment, so switching to propylthiouracil after confirming the diagnosis of AIT-1 with ^99m^-STS was the appropriate approach. There was significant improvement in his clinical condition afterward. This implies the importance of making the correct diagnosis in these patients to avoid unnecessary and potentially dangerous combined overtreatment. According to the European Thyroid Association 2018 guidelines, total thyroidectomy without delay is recommended in AIT patients with deterioration of the cardiac function or when they are unresponsive to the medical therapy [7]. Patient 1 had large MNG in the setting of deterioration of his medical condition, and he underwent total thyroidectomy. Improvement in the patient's clinical condition was noticed afterward.

## 5. Conclusion

^99m^-STS imaging is a valuable method for the diagnosis and classification of AIT. It can distinguish different types of AITs especially the mixed-types which are usually difficult to cure. ^99m^-STS has shown great advantages over the conventional methods. Further large prospective studies are required.

## Figures and Tables

**Table 1:** Summary of the patient's duration of amiodarone use and biochemical and radiological workup.

Patient	TSH	Free T4	Free T3	TRAb	TSI	Thyroid US	^99m-^STS	Duration of amiodarone use prior to presentation
Patient 1	<0.001	1.3	3.1	Negative	Negative	MNG	AIT-1	1 year
Patient 2	<0.01	5.06	6	Negative	Negative	Mild diffuse enlargement	AIT-1	1 year
Patient 3	0.06	2.3	2.6	Negative	Negative	MNG (multiple benign FNA)	AIT-1	1 year
Patient 4	0.01	1.75	3.2	Negative	Mildly positive	Not done	AIT-2	2 years
Patient 5	<0.004	3.4	5.72	Negative	Negative	Mild diffuse enlargement	AIT-1	18 months

**Table 2:** Summary of clinical, biochemical, and radiological imaging differentiation between AIT-1 and AIT-2 [1–19].

Modalities	AIT-1	AIT-2
Underlying thyroid disease	Yes (Graves' disease or MNG)	No
Time after starting amiodarone	Short (median 3 months)	Long (median 30 months)
Thyroid antibodies including TSI, TRAb, microsomal TPO antibodies	May be positive	Usually absent
RAIU	Low/normal/increased (uptake can be inhibited in high intrathyroidal iodine concentration and increased in iodine-deficient regions)	Low/absent
Circulating IL-6	Normal to high	Frequently marked elevated
T4/T3 ratio	Usually <4	Usually >4
Thyroid US	Diffuse or nodular goiter	Normal or small thyroid
CFDS	Increased	Absent
^99m^-STS	Increased uptake in the thyroid in the initial images followed by washout in the delayed images	Absent uptake in the thyroid in the initial and delayed images

**Table 3:** Summary of the different literature using ^99m^-STS in AIT.

Study	Type of the study	Number of patients	Country	Results
Piga et al. [17]	Prospective	20 patients	Italy	^99m^-STS can be proposed as an easy and highly effective diagnostic tool for the differential diagnosis of AIT, with positive persistent scans in AIT-1 and negligible uptake in AIT-2. It appears also to give some insights into indeterminate forms of AI. ^99m^-STS is superior to all other diagnostic tools including CFDS and RAIU.
Oki et al. [24]	Prospective	23 patients on amiodarone for more than 4 months (4 out of 23 patients had AIT)	Brazil	^99m^-STS may be an alternative tracer for thyroid scintigraphy and uptake measurement of patients on chronic use of amiodarone. ^99m^-STS seems to be better than Tc-99m pertechnetate for the scintigraphic evaluation of the thyroid in euthyroid and hyperthyroid patients.
Souto et al. [22]	Case report	1 patient	Portugal	^99m^-STS used to diagnosis undetermined AIT.
Pattison et al. [19]	Retrospective	15 patients	Australia	Use of quantitative TBR improves the interobserver reliability of reporting ^99m^-STS for investigation of different types of AIT.
Patel et al. [21]	Case report	1 patient	Houston	^99m^-STS used to diagnosis AIT-1 in addition to the RAIU.
Victor et al. [23]	Case report	1 patient	Portugal	^99m^-STS used to diagnosis undetermined AIT.
Wang and Zhang [18]	Prospective	15 patients	China	^99m^-STS imaging alone can accurately distinguish types I and II and mixed-type AIT.
Censi et al. [8]	Retrospective	30 patients	Italy	^99m^-STS combined with the TBR proved a very useful approach to the classification of AIT, enabling patients to be offered appropriate treatment as soon as they are diagnosed. It has 100% specificity and 91.7% sensitivity in differentiating different types of AIT.

## Abbreviation

AIH: Amiodarone-induced hypothyroidism
AIT: Amiodarone-induced thyrotoxicosis
AIT-1: Amiodarone-induced thyrotoxicosis type 1
AIT-2: Amiodarone-induced thyrotoxicosis type 2
AIT-3: AIT type 3
A fib: Atrial fibrillation
CRP: C-reactive protein
CFDS: Color flow Doppler sonography
DEA: Desethylamiodarone
ESR: Erythrocyte sedimentation rate
FNA: Fine-needle aspiration
IL-6: Interleukin-6
MNG: Multinodular goiter
PO: Orally
RAIU: Radioactive iodine uptake scan
TBR: Target to background ratio
TSH: Thyroid-stimulating hormone
T4: Thyroxine
T3: Triiodothyronine
TSI: Thyroid-stimulating immunoglobulin
TRAb: TSH receptor antibodies
TPO: Thyroid peroxidase
US: Ultrasound
VT: Ventricular tachycardia
^99m^-STS: ^99m^Tc-Methoxy-isobutyl-isonitrile (sestaMIBI) uptake on scintigraphy.
